# Does Concomitant Meniscectomy or Meniscus Repair Affect Muscle Strength, Lower Extremity Balance, and Functional Tests after Anterior Cruciate Ligament Reconstruction?

**DOI:** 10.3390/jcm13113310

**Published:** 2024-06-04

**Authors:** Maciej Biały, Kamil Kublin, Bartosz Wilczyński, Florian Forelli, Rafał Gnat

**Affiliations:** 1 Institute of Physiotherapy and Health Sciences, The Jerzy Kukuczka Academy of Physical Education, 40-065 Katowice, Poland; 2 Functional Diagnostics Laboratory, Sport-Klinika, Scanmed Sport, 44-240 Żory, Poland; 3 Motion Analysis Laboratory, Institute of Physiotherapy and Health Sciences, The Jerzy Kukuczka Academy of Physical Education, 40-065 Katowice, Poland; kublinkamil@gmail.com (K.K.); rafal.gnat@interia.pl (R.G.); 4 Department of Immunobiology and Environment Microbiology, Medical University of Gdańsk, 80-210 Gdańsk, Poland; bartosz.wilczynski@gumed.edu.pl; 5 Orthosport Rehab Center, Sport Rehabilitation Department, 95330 Domont, France; florian.forelli.mk@gmail.com; 6 Clinic of Domont, Education, Rehabilitation and Research Department, Orthopedic Surgery Department, Ramsay Healthcare, 95330 Domont, France; 7 SFMKS-Lab, Société Française des Masseurs-Kinésithérapeutes du Sport, 93380 Pierrefitte-sur-Seine, France

**Keywords:** anterior cruciate ligament, reconstruction, meniscal repair, meniscectomy, functional performance

## Abstract

**Background/Objective**: The effects of concomitant meniscal tears and their associated treatment on strength, lower extremity balance, and functional status after anterior cruciate ligament reconstruction (ACLR) have not been widely investigated. This study aimed to compare the functional outcomes in patients who underwent ACLR with concomitant treatment of the medial meniscus repair versus meniscectomy when returning to unrestricted physical activity. **Methods**: A total of 85 patients who underwent primary ACLR with combined meniscal repair (MREP; *n* = 39) or meniscectomy (MRES; *n* = 46) were assessed. The dataset included the Functional Movement Screen^TM^ (FMS) outcomes and single-leg balance test (SLBT) with anterior–posterior, medial–lateral, and overall stability indexes. Isokinetic knee extension and flexion strengths were tested at velocities of 60 deg·s^−1^ and 180 deg·s^−1^. The peak torque-to-body weight ratio (PT/BW) and limb symmetry index (LSI) were calculated. **Results**: In the functional assessment, there was no significant inter-group difference in the composite score of the FMS (MREP: 15.08 pts vs. MRES: 15.13 pts; *p* > 0.05). The SLBT outcomes in inter-group and inter-extremity comparisons were irrelevant (*p* > 0.05), too. Significant differences emerged in the inter-group comparison of the knee extension strength in the non-operated extremity at both 60 deg·s^−1^ and 180 deg·s^−1^ (*p* = 0.02). Inter-extremity differences were significant in both the MREP and MRES groups for knee extension and flexion at both angular velocities (all *p* values < 0.05). For knee extension, the LSI values ranged from 82% to 87%, and for flexion, from 77% to 84%, with no significant inter-group differences. **Conclusions**: Patients undergoing ACLR with concomitant meniscal repair or resection did not exhibit differences in isokinetic muscle strength, lower extremity balance, and functional tests upon returning to activity. However, participants in both groups demonstrated significant differences between the operated and non-operated extremities as far as the knee joint extensor and flexor strengths are concerned. Therefore, rehabilitation protocols should prioritize equalizing inter-extremity strength differences after the ACLR with additional treatment procedures addressing the menisci.

## 1. Introduction

An anterior cruciate ligament (ACL) tear is frequently associated with concomitant injuries to other structures of the knee joint, including articular cartilage, collateral ligaments, and the menisci [1,2,3]. Medial meniscus tears are observed in more than 60% of individuals with ACL tears, while lateral meniscus tears occur in 30% [2,4]. As previously reported, the meniscus plays a crucial role in knee laxity, load distribution, and proprioception [3,5]. Consequently, damage to the meniscus may jeopardize the long-term outcomes of ACL reconstruction (ACLR) [6]. In patients with ACL rupture, an associated meniscus injury may necessitate modifications to the postoperative rehabilitation program and can influence functional gains following surgical intervention [5,7]. While the outcomes of ACLR have been well documented [4,5,6,8,9], investigators are still trying to determine the most suitable way to manage meniscal lesions during primary ACLR [2,6,10]. The guidelines for managing meniscal injuries remain, however, uncertain [11]. For meniscal tears, the most likely options are either to repair the tear with suturing or to resection the damaged tissue [2,3,5]. Recent studies have questioned the value of a meniscectomy during isolated meniscal injury, as meniscal repair can result in better outcomes and an improved laxity score [5,12,13]. Moreover, there is a lack of data confronting functional outcomes of medial meniscal repair versus meniscectomy with concomitant ACLR [2,5,6,12].

The ACL deficiency contributes to the knee joint degeneration, as indicated by various studies [7,11,14]. Recent research has aimed to elucidate the impact of meniscal repair or resection on postoperative outcomes following ACLR [2,6,10,15]. Numerous authors report that rates of osteoarthritis after combined ACL and meniscal injuries range between 21–48% in the minimum 10-year follow-up [5,6,11]. Medial or lateral meniscus resections emerge as significant risk factors for knee osteoarthritis in a 14-year follow-up [16]. At the same time, meniscal repair does not seem to accelerate the degeneration of the knee joint [6].

It is worth noting that most ACLR patients exhibit strength/motor control deficits in the lower extremity and core region when attempting to resume unrestricted sports activities [17,18,19,20,21,22]. Only 63% of individuals achieve their pre-injury sports level [21,22]. These persons very often exhibit the side-to-side differences between the operated (OP) and non-operated (NOP) extremities during functional activities like single-leg jumps or impaired kinematics during multi-joint motor tasks like bilateral countermovement jumps or squats, which is not observed in healthy individuals [18,19]. Additionally, limited data are available on the impact of the concomitant ACL/meniscal injuries on the ability to regain functional symmetry between extremities after ACLR [2,3,5]. While investigators suggest that a functional evaluation after ACLR should be grounded in objective criteria [8,23], there is still a lack of consensus regarding the standardized and objective test protocols ensuring a safe return to pre-injury training loads [2,11,15].

This study aimed to evaluate functional outcomes in two groups of patients: the first, undergoing ACLR with medial meniscal repair, and the second, undergoing ACLR with partial medial meniscal resection. Furthermore, we assessed muscle strength and neuromuscular control imbalances between the OP and NOP extremities. 

## 2. Materials and Methods

### 2.1. Study Design

This is a prospective cohort study comparing two groups of patients: one underwent medial meniscal repair (MREP) and the other underwent partial medial meniscal resection (MRES), alongside simultaneous ACLR using hamstring tendon autograft. The research was conducted in agreement with the Declaration of Helsinki. It was approved by the local Research Ethics Committee (No. 2017/3). All participants provided their written informed consent. The functional assessment encompassed the Functional Movement Screen™ (FMS), single-leg balance test (SLBT), and isokinetic strength tests. These evaluations were conducted when subjects were on the verge of returning to unrestricted activity. In the MREP group, this was (on average) 7.13 ± 3.28 months post-ACLR, and in the MRES group, 6.33 ± 2.84 months post-ACLR.

### 2.2. Population

Eighty-five patients who underwent primary anterior ACLR with concomitant medial meniscus repair (MREP: *n* = 39) or resection (MRES: *n* = 46) were recruited. The patients were recruited by one surgeon with 15+ years of experience in ACL surgery. All participants met the inclusion criteria: diagnosis of ACL rupture with concomitant medial meniscus tear confirmed using magnetic resonance imaging, followed by ACLR with simultaneous management of the medial meniscus tear by either (1) repair or (2) resection, based on an intraoperative decision. Participants were excluded if they had any history of prior ACL reconstruction to prevent confounding effects from previous surgeries. Those with previous meniscus surgeries, including repairs or resections, were also excluded to focus solely on the impact of the ACL condition. Additionally, individuals who underwent other joint surgeries during their ACL treatment—such as posterior lateral complex reconstruction, lateral meniscus repair or resection, or articular cartilage repair—were not included. Furthermore, participants experiencing pain or discomfort in the musculoskeletal system, as indicated by a score above zero on the Visual Analogue Scale (VAS) at the time of measurement, were excluded to ensure the accuracy and relevance of the study findings. None of the participants underwent preoperative rehabilitation. Both groups followed the rehabilitation program based on the protocol proposed by Lepley et al. [24]. Each patient received detailed guidelines for rehabilitation in the early postoperative phase. This included emphasizing the knee range of motion, edema management, muscle strength drills, and exercise progression regimens.

### 2.3. Raters

All measurements were conducted by the two certified physiotherapists, each with ten years of orthopedic rehabilitation experience and five years of specialized training in functional testing. To ensure objectivity, a third independent rater, a professor with academic expertise, who was unaware of the objective of the study processed the data. Measurement sequences were randomized prior to the study and consistently applied to each subject in the following order: FMS, SLBT, and isokinetic strength tests. Additionally, the extremity initiating the measurements during the SLBT and strength tests was randomized. The reliability of the measurements was established in the group of 12 healthy subjects. The weighted Kappa–Kohen coefficient of 0.75 was obtained for FMS. Additionally, intra-rater reliability was evaluated, showing the intra-class correlation coefficient (ICC) of 0.88 for SLBT and 0.94 for the peak torque/body weight ratio calculated in the isokinetic strength tests. Based on the established criteria, ICC values <0.5, 0.5–0.75, 0.75–0.9, and >0.90 denote poor, moderate, good, and excellent reliability, respectively [25]. The presented reliability indices align with previous data reported by other authors [26,27,28,29].

### 2.4. Functional Assessment

Before conducting the examination, each patient underwent a 10 min warm-up session on a bicycle ergometer. During the warm-up, the rater briefly explained each step of the functional assessment procedure and inquired about the patient’s health condition, particularly the absence of any current pain symptoms within the musculoskeletal system. Patients were encouraged to communicate any discomfort or pain experienced during the examination, and they were free to terminate the tests at any stage.

The functional assessment began with the standardized FMS tests. This battery comprises seven dynamic tests to evaluate the quality of fundamental movement patterns based on factors such as muscle strength, flexibility, range of motion, and neuromuscular control. These tests help detect major motor dysfunction or asymmetries [30]. Each participant completed three trials of each of the seven FMS tests (the mean value of the three was considered during subsequent data analysis), including the deep squat, in-line lunge, hurdle step, shoulder mobility, active straight leg raise, trunk stability push-up, and quadruped rotary stability [30]. Participants scored from 0 to 3 points for each test using specific criteria. A score of 0 indicated the presence of pain during the movement; 1, the inability to complete the given motor task; 2, the completion of the task with additional compensatory movements; and 3, efficient completion of the movement. The maximum achievable total score was 21 points [30]. The FMS has been reported to be a reliable tool for musculoskeletal screening [26].

The SLBT was conducted using the Biodex Balance System (Biodex Medical Systems, Shirley, NY, USA), which utilizes sensors beneath its platform to measure the deviation of the patient’s center of pressure from the center of the platform. The assessment included static (platform locked in a horizontal position) and dynamic (platform tilted up to a maximum of 20 degrees from the horizontal position in all directions) conditions. Dynamic measurements may be performed at various difficulty levels, ranging from level 1 (most difficult/least stable) to level 12 (easiest/most stable). For safety reasons, the platform in this study was set at level 4 for both groups. The SLBT comprised three types of output measures: anterior–posterior (AP), medial–lateral (ML), and overall (OSI) stability indexes. These indices represent the standard deviations of the angle of inclination of the platform relative to the horizontal plane. In contrast, in static tests, the device calculates the projection of the subject’s center of pressure on the platform. Subsequently, it determines the presumed angle of platform inclination based on the given center of pressure position. A lower score indicates less variability in sway and therefore is more desirable [31]. The OSI, AP, and ML were calculated using the formula previously described by Arnold and Schmitz [31], which has been reported as reliable [27,28]. During the measurement, each participant was instructed to assume the one-legged standing position on the circular platform with the contralateral extremity flexed to 90 degrees at the knee/hip joints. Each subject performed three repetitions of the one-legged stance for 20 s without visual feedback [27]. The mean value of the trials was used for inter-group and inter-extremity analyses.

In the last phase of the procedure, after a 5 min rest interval, quadriceps and hamstring muscle strength measurements were assessed using the Biodex System 4 (Biodex Medical Systems, Shirley, NY, USA). Isokinetic knee flexion and extension peak torques were evaluated at angular velocities of 60 deg·s^−1^ and 180 deg·s^−1^. Each participant was seated on the dynamometer’s seat and secured with belts around their trunk, pelvis, and thighs. The resistance pad was positioned at the level of the medial malleolus of the tested extremity. A one-minute rest interval was introduced between each trial. For each muscle group at each angular velocity, the peak torque normalized by the body weight (PT/BW ratio [Nm/kg]) was calculated [24,32]. Isokinetic strength tests have demonstrated high test–retest reliability (ICC from 0.81 to 0.97) in patients after ACLR [29]. Additionally, for knee flexion and extension at both angular velocities, the limb symmetry indexes (LSIs) were computed using the formula: LSI = (peak torque for injured leg/peak torque for uninjured leg) × 100 [3]. 

### 2.5. Statistical Methods

In the data analysis, each lower extremity was considered the separate statistical unit categorized as either the OP or NOP. To compare inter-group differences (MREP vs. MRES) and inter-extremity differences, either the independent Student’s *t*-test (normal data distribution) or Mann–Whitney U test (non-normal data distribution) were utilized. The normality of data distributions was assessed using the Shapiro–Wilk test. A significance threshold of *p* < 0.05 was applied for all comparisons. Statistica software 13.3 (Statistica, Tulsa, OK, USA) was employed.

## 3. Results

### 3.1. Participant Characteristics

In the MREP group, out of 39 subjects, 15 were female, whereas in the MRES group, out of 46 subjects, 17 were female. The mean age (± standard deviation), body height, and body mass for the MREP group were as follows: 25.56 (±7.2) years, 172.36 (±18.38) cm, and 73.08 (±12.34) kg, respectively. Correspondingly, for the MRES group, these values were as follows: 29.75 (±13.07) years, 176.06 (±9.73) cm, and 76.11 (±13.82) kg, respectively (see Table 1).

### 3.2. Functional Assessment

In the functional assessment, there were no significant inter-group differences in the total score of the FMS test. The mean score (± standard deviation) in the MREP group was 15.08 (±2.38) points, and in the MRES group, it was 15.13 (±2.30) points. No significant differences were found (*p* < 0.05). 

**Figure 1:**
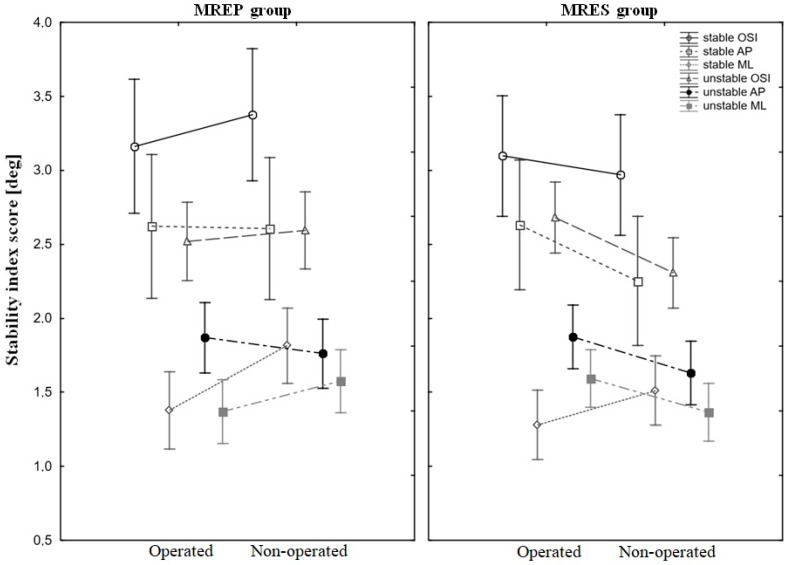
Mean values and 95% confidence intervals (whiskers) of the outcomes of all balance tests. Balance tests were performed on stable and unstable surfaces with operated and non-operated extremities. OSI—overall stability index, AP—anterior–posterior, ML—medial–lateral, MREP—medial meniscus repair, MRES—medial meniscus partial resection.

The results of the SLBT in the inter-group and inter-extremity comparisons were mainly non-significant (Table 2). However, some irregular exceptions occurred. The inter-extremity difference in the ML index on the stable surface in the MREP group was significant (*p* = 0.01), with recorded values of 1.34 (±0.7) for the OP extremity and 1.83 (±0.9) for the NOP extremity. Moreover, the inter-extremity difference in the OSI on the unstable surface in the MRES group was significant (*p* = 0.04), with an OSI equal to 2.75 (±1.06) for OP and 2.54 (±0.85) for NOP extremities. The intra-group difference in the ML index on the unstable surface was also significant (*p* = 0.04), with values of 1.63 (±0.72) for NOP extremity in the MREP group and 1.34 (±0.45) in the MRES group. Mean values and 95% confidence intervals of all SLBTs are presented in Figure 1.

**Figure 2:**
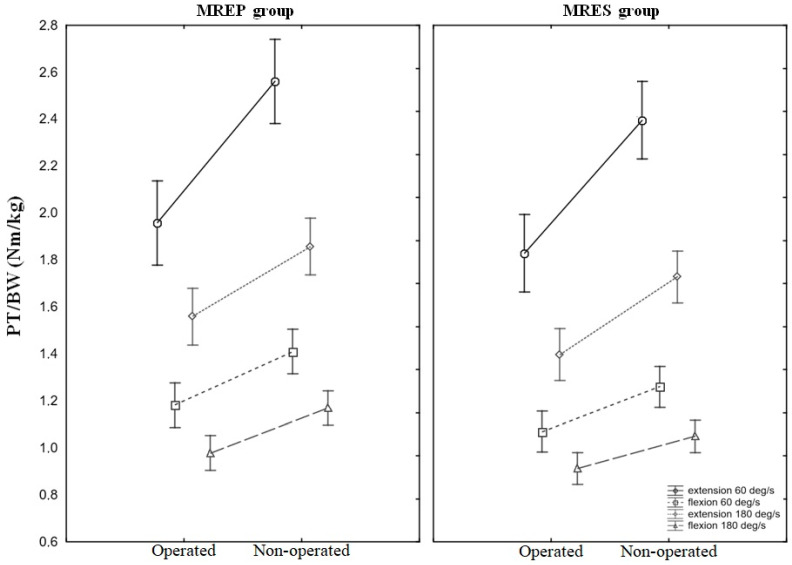
Mean values and 95% confidence intervals (whiskers) of the outcomes of all isokinetic tests. PT/BW ratios were recorded at the angular velocities of 60 deg·s^−1^ and 180 deg·s^−1^ with operated and non-operated extremities. MREP—medial meniscus repair, MRES—medial meniscus partial resection.

The isokinetic tests exhibited a more regular pattern (Table 2). Inter-group comparisons revealed significant outcomes for knee extension of the NOP extremity at both angular velocities of 60 deg·s^−1^ and 180 deg·s^−1^. PT/BW ratios were significantly higher in the MREP group, with values of 2.56 (±0.56) vs. 2.39 (±2.39) Nm/kg (*p* = 0.02) and 1.86 (±0.39) vs. 1.73 (±0.37) Nm/kg (*p* = 0.02), respectively. The inter-extremity differences between OP and NON were significant in both the MREP and MRES groups at both angular velocities and for both muscle groups. In the MREP group, for an angular velocity of 60 deg·s^−1^, the quadriceps PT/BW ratios for OP and NOP extremities were 1.96 (±0.58) vs. 2.56 (±0.56) Nm/kg (*p* < 0.0000) and 1.18 (±0.28) vs. 1.41 (±0.29) Nm/kg (*p* < 0.0008) for hamstring PT/BW ratios. For an angular velocity of 180 deg·s^−1^, the values were 1.56 (±0.37) vs. 1.86 (±0.39) Nm/kg (*p* < 0.0008) and 0.98 (±0.20) vs. 1.17 (±0.24) Nm/kg (*p* < 0.0002), respectively. In the MRES group, for an angular velocity of 60 deg/s, the PT/BW ratios for OP and NOP extremities were 1.83 (±0.61) vs. 2.39 (±0.52) Nm/kg (*p* < 0.0000) and 1.07 (±0.28) vs. 1.26 (±0.30) Nm/kg (*p* < 0.004) for hamstring PT/BW ratios. For an angular velocity of 180 deg·s^−1^, these values were 1.40 (±0.41) vs. 1.73 (±0.39) Nm/kg (*p* < 0.0001) and 0.91 (±0.25) vs. 1.05 (±0.23) Nm/kg (*p* < 0.008), respectively. The mean values and 95% confidence intervals of all PT/BW ratios are presented in Figure 2.

The LSI results for both the MREP and MRES groups exhibited very similar outcomes. For knee extension at 60 deg·s^−1^, the LSI results for both groups were 77 (±16.91) vs. 77 (±15.79)% (*p* = 0.74). Similarly, for knee flexion at the same velocity, the LSI results for both groups were 82 (±13.49) vs. 85 (±14.47)% (*p* = 0.35). At the velocity of 180 deg·s^−1^, the LSIs for knee extension were 84 (±15.38) vs. 81 (±14.37)% (*p* = 1.13) for MREP and MRES, respectively. For knee flexion, the LSIs were 84 (±12.88) vs. 87 (±13.7)% (*p* = 0.09), respectively (see Table 3).

## 4. Discussion

The main finding of this study is the similarity in the postoperative functional outcomes between patients who underwent ACLR with simultaneous MREP or MRES. The results did not significantly differ between the two groups across all measured variables, including FMS, SLBT, and muscle strength tests. The only distinction was the inter-extremity difference in strength, with the operated extremity proving to be significantly weaker in both MREP and MRES groups. Therefore, by comparing the functional outcomes between the two groups, it is difficult to determine which approach to meniscal tears management is more beneficial for patient.

The evaluation of post-ACLR functional outcomes based on FMS tests yielded conflicting findings. In this study, no significant difference was observed in the total FMS score between the MREP (15.08 ± 2.38 points) and MRES (15.13 ± 2.30 points) groups. This aligns with the findings of Mayer et al. [20], who found no significant difference in the FMS total scores between ACLR patients cleared for unrestricted sporting activities (12.7 ± 2.9 points) and those not cleared (12.8 ± 2.7 points) six months post-surgery [20]. However, as compared to Mayer et al. [20], Biały et al. reported better FMS outcomes (14.7 ± 2.4) in ACLR patients evaluated 3–4 months post-surgery [33]. It is worth noting that the high FMS total score might lead to an overestimation of functional outcomes, prompting additional tools (e.g., jump tests) to provide a more comprehensive assessment of the patient [20,33]. Despite similar FMS results in the MREP and MRES groups, caution is advised in interpreting these outcomes. 

Previous studies have indicated that patients undergoing ACLR, meniscal repair, and resection often exhibit an impaired single-leg stance and postural control [19,34,35]. However, no significant differences in the SLBT were found for OP extremities between the MREP and MRES groups in this study. Nonetheless, upon analyzing the SLBT results in the MREP group, it was noted that nearly all SLBT ratios favored the OP extremity (except the AP values on the unstable surface: 1.91 vs. 1.82), contrasting with the findings in the MRES group. Here, the NOP extremity exhibited superior balance compared to the OP (except for the ML values on the stable surface: 1.30 vs. 1.55), and the OSI value was notably lower for the NOP on the unstable surface (OP = 2.75 vs. NOP = 2.45). While no inter-group differences were observed, a trend emerged, suggesting that postural control on the OP extremity was more effective in the MREP group across both stable and unstable surfaces. This trend may align with data from Sarraj, who demonstrated that meniscal repair leads to decreased anterior knee joint laxity and improved patient-reported outcomes [5].

Regarding muscle strength, the study found no differences between the operated extremities in both groups, as evidenced by similar quadriceps and hamstring PT/BW ratios. However, across both groups, all recorded LSI values, irrespective of group assignment, angular velocity, or muscle group, fell below 90%. This observation is consistent with Senorski et al.’s [3] findings, who reported that less than one in four patients undergoing ACLR with concurrent meniscal tears achieved an LSI of ≥90% one year post-surgery. These LSI outcomes hold significance for determining the time of return to sports or activities, ensuring a safe return to preoperative training intensity and minimizing the risk of subsequent injuries [3]. The results of the strength tests in this study align with findings by Leplay et al. [24], indicating no inter-group disparities in quadriceps strength among patients undergoing partial medial meniscectomy or repair alongside ACLR. However, differences were observed within the MREP and MRES groups, revealing that participants generated notably higher peak torques with the NOP knee as compared to the OP knee. This trend emphasizes the challenge of fully restoring the quadriceps strength to preoperative levels following ACLR, as highlighted in the literature [6,8,18]. Lower results in isokinetic strength tests may signify incomplete quadriceps recovery, posing a potential risk factor for subsequent ACL re-injuries [7,20].

Our findings regarding the overall functional status of patients align with previous research by LaPrade et al. [6], who reported similar outcomes and observed no significant differences in the two-year follow-up between patients undergoing isolated ACLR and those undergoing ACLR with medial or lateral meniscal resection or repair, as evidenced by Knee Injury and Osteoarthritis Outcome Score (KOOS) subscales in the Norwegian Knee Ligament Registry. However, Phillips et al. [2] presented contrasting data, indicating that ACLR with concurrent meniscus resection resulted in a poorer clinical outcome than isolated ACLR. Interestingly, this correlation was not observed in the meniscus repair group. This suggests that whenever feasible, meniscus repair may yield superior clinical outcomes as compared to meniscal resection. The study by Phillips et al. was in fact a retrospective cohort analysis based on self-reported questionnaires (KOOS, Activities of Daily Living Scale (ADLS), Quality of Life Scale (QOLS), and Visual Analogue Scale (VAS)) [2]. In contrast, Sarraj et al. [5] noted that patients undergoing ACLR combined with meniscus resection demonstrated improved symptoms in the two-year follow-up as compared to those undergoing ACLR plus meniscus repair. Sarraj et al. observed that ACLR plus meniscus repair led to reduced anterior knee joint laxity (measured with a KT-1000 arthrometer) and improved patient-reported outcomes in the long term, but these ‘gains’ were associated with higher re-operation rates.

The presented study has several limitations that should be acknowledged. First, preoperative functional outcomes, including FMS scores, SLBT results, and the muscle strength status, were not recorded, which could have provided valuable baseline data for comparison. Second, the rehabilitation protocols differed slightly between the MREP and MRES groups during the early postoperative phase, potentially introducing variability in the recovery process and minor discrepancies in time interval from the surgery to measurement. Additionally, the study did not analyze potential differences between genders, and the findings may need to be generalizable to high-level athletes, as the study was conducted only on a physically active general population. Moreover, it should be noted that lacking determination of the meniscal tear type is also to be considered a study limitation. Furthermore, future investigations could explore differences between subtypes of medial meniscus tears, as not all tears are repairable, and the type of tear may influence treatment decisions and outcomes. Long-term follow-ups, 15 years and more, would also be valuable for assessing the durability and efficacy of different meniscal treatment methods combined with ACLR. This research field remains relatively understudied and warrants further investigation to better inform clinical practice and optimize patient outcomes.

## 5. Conclusions

No significant differences between the two investigated surgical approaches were found. This conclusion applies to the functional outcomes obtained in the study, i.e., the FMS and SLBT, as well as the lower extremity muscle strength tests. It remains unclear which management option (meniscus repair vs. resection) exerts more significant influence on the postoperative functional outcomes. This notion underlines the multifaceted nature of individual responses to treatment and the importance of personalized clinical decision making.

Significant weakness in the operated extremity was observed as well. This was consistent across all isokinetic tests (regardless of surgical intervention type, angular velocity, and direction of movement). 

## Figures and Tables

**Table 1:** Homogeneity of the MREP and MRES groups. Data are reported as mean values ± standard deviations.

	Group MREP (*n* = 39)	Group MRES (*n* = 46)	*p*-Value
Gender (male; female)	M: 24; F: 15	M: 29; F: 17	0.85 ***
Body mass (kg)	73.08 ± 12.34	76.11 ± 13.82	0.30 *
Body height (cm)	172.36 ± 18.38	176.06 ± 9.73	0.20 *
Age at operation time (y)	25.56 ± 7.2	29.75 ± 13.07	0.09 **
Trauma–operation interval (mth)	11.59 ± 14.75	14.44 ± 12.71	0.17 **
Operation–measurement interval (mth)	7.13 ± 3.28	6.33 ± 2.84	0.23 **

* Independent Student’s *t*-test; ** Mann–Whitney *U* test; *** Chi^2^ test.

**Table 2:** Mean values ± standard deviations along with minimal–maximal values for the outcomes of all balance tests and isokinetic tests were calculated. The balance tests were conducted on both stable and unstable surfaces. Muscle strength ratios were recorded at angular velocities of 60 deg·s^−1^ and 180 deg·s^−1^. The results include *p*-values for both inter-group and inter-extremity comparisons.

		MREP Group			MRES Group			*p*-Value MRES vs. MREP for OP	*p*-Value MRES vs. MREP for NOP
Parameter		OP	NOP	*p*-Value OP vs. NOP	OP	NOP	*p*-Value OP vs. NOP		
Balance Tests									
Stable surface	OSI	3.14 ± 1.01 (2.81–3.47)	3.47 ± 1.50 (2.99–3.96)	0.25 **	3.23 ± 1.77 (1.00–7.70)	2.99 ± 0.87 (1.20–5.60)	0.42 *	0.78 **	0.07 *
	AP	2.54 ± 1.12 (2.18–2.91)	2.62 ± 1.48 (2.14–3.10)	0.78 *	2.72 ± 1.91 (0.70–7.74)	2.34 ± 0.68 (0.60–6.20)	0.21 **	0.61 *	0.25 **
	ML	1.34 ± 0.70 (1.11–1.57)	1.83 ± 0.90 (1.53–2.12)	0.01 **	1.30 ± 0.58 (0.50–2.60)	1.55 ± 0.74 (0.34–3.50)	0.09 **	0.77 **	0.12 **
Unstable surface	OSI	2.62 ± 0.99 (2.29–2.95)	2.75 ± 1.04 (2.40–3.10)	0.56 *	2.75 ± 1.06 (1.40–5.60)	2.45 ± 0.85 (1.10–5.30)	0.04 **	0.56 **	0.15 **
	AP	1.91 ± 0.73 (1.66–2.15)	1.82 ± 0.74 (1.58–2.07)	0.57 **	1.85 ± 1.30 (0.70–3.6)	1.68 ± 0.69 (0.57–3.69)	0.44 *	0.80 **	0.37 *
	ML	1.39 ± 0.66 (1.17–1.61)	1.63 ± 0.72 (1.39–1.87)	0.11 *	1.61 ± 0.86 (0.60–3.30)	1.34 ± 0.45 (0.32–2.40)	0.07 *	0.20 *	0.04 *
Isokinetic tests (Nm/kg)									
PT/BW at 60 deg·s^−1^	ext.	1.96 ± 0.58 (1.77–2.14)	2.56 ± 0.56 (2.38–2.74)	<0.0000 *	1.83 ± 0.61 (0.59–3.16)	2.39 ± 0.52 (1.40–3.48)	<0.0000 *	0.32 *	0.02 *
	flex.	1.18 ± 0.28 (1.09–1.27)	1.41 ± 0.29 (1.31–1.51)	0.0008 *	1.07 ± 0.31 (0.46–1.75)	1.26 ± 0.30 (0.58–2.07)	0.004 *	0.09 *	0.06 *
PT/BW at 180 deg·s^−1^	ext.	1.56 ± 0.37 (1.44–1.68)	1.86 ± 0.39 (1.73–1.98)	0.0008 *	1.40 ± 0.41 (0.44–2.19)	1.73 ± 0.37 (0.90–2.44)	0.0001 *	0.06 *	0.02 *
	flex.	0.98 ± 0.20 (0.91–1.04)	1.17 ± 0.24 (1.09–1.25)	0.0002 *	0.91 ± 0.25 (0.44–1.53)	1.05 ± 0.23 (0.48–1.67)	0.008 *	0.16 *	0.05 *

* Independent Student’s *t*-test; ** Mann–Whitney *U* test. OP—operated extremity, NOP—non-operated extremity, OSI—overall stability index, AP—anterior–posterior, ML—medial–lateral, PT/BW—peak torque/body weight ratio, ext.—extension, flex.—flexion.

**Table 3:** Mean values ± standard deviations for limb symmetry index values (%) for tested groups. Relative quadriceps femoris and hamstring muscle strength ratios were recorded at angular velocities of 60 deg·s^−1^ and 180 deg·s^−1^. All *p*-levels for Mann–Whitney U test.

		MREP Group (%)	MRES Group (%)	*p*-Value
PT/BW 60 deg·s^−1^	ext.	77 ± 16.91	77 ± 15.79	0.98
	flex.	82 ± 13.49	85 ± 14.47	0.31
PT/BW 180 deg·s^−1^	ext.	84 ± 15.38	81 ± 14.37	0.34
	flex.	84 ± 12.88	87 ± 13.7	0.29

PT/BW—peak torque/body weight ratio, ext.—extension, flex.—flexion.

## Data Availability

The data presented in this study are available on request from the corresponding author. The data are not publicly available due to privacy restrictions.
